# BICORN: An R package for integrative inference of *de novo* cis-regulatory modules

**DOI:** 10.1038/s41598-020-63043-2

**Published:** 2020-05-14

**Authors:** Xi Chen, Jinghua Gu, Andrew F. Neuwald, Leena Hilakivi-Clarke, Robert Clarke, Jianhua Xuan

**Affiliations:** 1 Bradley Department of Electrical and Computer Engineering, Virginia Polytechnic Institute and State University, 900 North Glebe Road, Arlington, VA 22203 USA; 2 Baylor Research Institute, 3310 Live Oak St, Dallas, TX 75204 USA; 3 Institute for Genome Sciences and Department Biochemistry & Molecular Biology, University of Maryland School of Medicine, Baltimore, MD 21201 USA; 4 Department of Oncology, Lombardi Comprehensive Cancer Center, Georgetown University Medical Center, 3970 Reservoir Road, Washington, DC 20057 USA

**Keywords:** Cancer genomics, Gene regulatory networks

## Abstract

Genome-wide transcription factor (TF) binding signal analyses reveal co-localization of TF binding sites based on inferred cis-regulatory modules (CRMs). CRMs play a key role in understanding the cooperation of multiple TFs under specific conditions. However, the functions of CRMs and their effects on nearby gene transcription are highly dynamic and context-specific and therefore are challenging to characterize. BICORN (Bayesian Inference of COoperative Regulatory Network) builds a hierarchical Bayesian model and infers context-specific CRMs based on TF-gene binding events and gene expression data for a particular cell type. BICORN automatically searches for a list of candidate CRMs based on the input TF bindings at regulatory regions associated with genes of interest. Applying Gibbs sampling, BICORN iteratively estimates model parameters of CRMs, TF activities, and corresponding regulation on gene transcription, which it models as a sparse network of functional CRMs regulating target genes. The BICORN package is implemented in R (version 3.4 or later) and is publicly available on the CRAN server at https://cran.r-project.org/web/packages/BICORN/index.html.

## Introduction

Transcription factor (TF)-DNA binding profiles are widely available since the rapid development of epigenetic biotechnologies^1,2^. TFs regulate gene expression by binding at regulatory non-coding area (promoters or enhancers) and thereby recruiting RNA polymerase II and co-factors required for gene transcription^3^. A cis-regulatory module (CRM) is defined as a stretch of DNA with binding sites of multiple TFs and has been frequently observed at active promoter or enhancer regions^4,5^. Jointly modelling associations of multiple TFs at regulatory regions helps to uncover synergy among TFs and their regulatory effects on gene transcription^6^.

TF-associations (as CRMs) can be identified from genome-wide binding sites of individual TFs, their co-binding events, and their effects on gene transcription^7–9^. A major limitation of existing packages for inferring genome-wide *de novo* CRMs^10,11^ is that they do not integrate with gene expression data and thus, cannot predict the regulatory effects of CRMs on genes. Conventional TF-gene regulatory network inference tools^12–14^ fail in this regard either because they focus on individual TF-gene interactions rather than on multiple TFs associations. Among those tools focusing on the regulatory potential of multiple TFs, most only report strong regulators for each gene in order to avoid overfitting—which leads to incomplete regulatory network reconstruction.

To address these issues, here we describe BICORN (Bayesian Inference of COoperative Regulatory Network), a tool for functional CRM inference. Given TF-gene binding observations, BICORN first identifies a list of candidate CRMs, each with a unique combination of TFs. Next, for each CRM, target gene expression is modeled as a log-linear combination of TF activities and gene regulation strengths. Using Gibbs sampling, BICORN iteratively estimates TF activity, TF-gene regulatory strengths, and the posterior distribution of corresponding CRM models. Notably, for TFs in each CRM, their regulation strengths on a target gene are modeled jointly, so that sampling is based on the CRM’s overall regulatory effect on target gene transcription. For each CRM, a small number of TFs are jointly represented so that overfitting effects are largely alleviated and both strong and weak regulators (if any) on each gene are captured. (BICORN is named after a mythical beast composed of a combination of creatures, because it models CRMs composed of a combination of TFs.).

To support our hypothesis that TFs often regulate gene expression synergistically by forming CRMs rather than independently, we applied BICORN to multiple simulated and publicly available benchmark data sets. The accuracy and robustness of BICORN is indeed higher than that of conventional regulatory network inference tools. We further used BICORN to infer CRMs from diverse cell types and found that, for the same cell type (i.e. breast cancer MCF-7 cells), TFs function and associate differently at enhancers than at promoters and have associations that differ among different cell types. Therefore, BICORN-inferred CRMs are accurate and cell type-specific. We implemented BICORN in a R package and made it publicly available on the CRAN server at https://cran.r-project.org/web/packages/BICORN/index.html.

## Methods

### BICORN input

**Figure 1:**
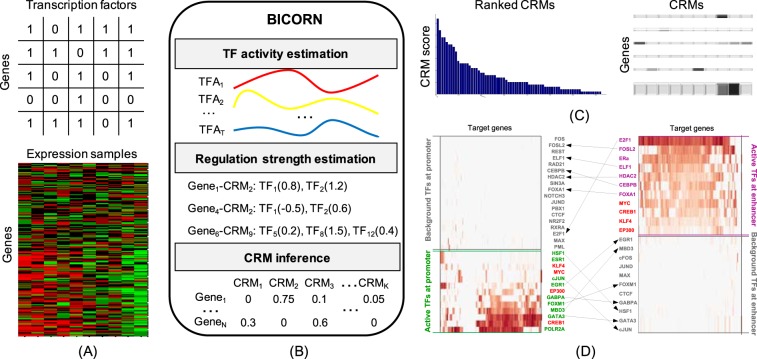
The workflow of BICORN. (**A**) For a particular cell type or tissue, given TF-gene binding events observed at promoter or enhancer regions and target gene expression data, (**B**) BICORN iteratively estimates TF activity (TFA), regulation strength (RS) and cis-regulatory modules (CRMs) using Gibbs sampling. (**C**) Based on the sampling statistics, a ranked list of CRMs (TF-associations) is obtained with target genes inferred for each. (**D**) Meanwhile, a TF-gene regulatory network is created so that TFs and their associations functioning under different situations (i.e., promoter vs. enhancer) can be compared.

BICORN requires two inputs: TF-gene binding events and gene expression data from the same context (Fig. 1A). Binary TF-gene binding input is used because it is the signal format most commonly used by different resources. For example, many databases (i.e., TRANSFACT) provided candidate target genes for a query TF; using ChIP-seq data, peaks of each TF can be aligned to enhancer or promoter regions and further associated with genes; for ATAC-seq data, TF motif enrichment scanning at accessible chromatin regions with gene annotation is a straightforward way to identify genes targeted by enriched TFs^15^.

To infer CRMs, BICORN identifies a list of candidate CRMs (in total *K*) for *T* given TFs. To account for prior binding knowledge, BICORN defines a candidate CRM matrix ***B*** with *K* rows and *T* columns. Each row $k\in \{1,2,\mathrm{..}.,K\}$ is a unique vector $[{b}_{k,1},{b}_{k,2},\mathrm{..}.,{b}_{k,T}]$ including binary binding states ${b}_{k,t}$ (1 or 0). To account for ‘background’ genes not regulated by any CRMs, an all-zero vector $[{b}_{0,1},{b}_{0,2},\mathrm{..}.,{b}_{0,T}]$ is included in ***B***. For gene *n*, BICORN defines a CRM index variable *c*_*n*_ (${c}_{n}\in \{1,2,\mathrm{..}.,K\}$) pointing to a row in ***B***, denoting the CRM regulating expression of gene *n*.

For gene expression data, RNA-seq data (transcripts per million (TPM) values) are preferred^16^ and microarray data can also be used with proper normalization. Given *M* expression profiles (samples) of *N* genes, we defined a gene expression matrix **Y**′ with each unit $y{\text{'}}_{n,m}$ ($n\in \{1,2,\mathrm{..}.,N\}$, $m\in \{1,2,\mathrm{..}.,M\}$) representing the expression value of gene *n* under sample *m*.

### BICORN algorithm

#### Log-linear model

Gene expression are usually measured and studied with respect to a reference level. Thus, for gene *n*, we approximate its expression $y{\text{'}}_{n,m}$ by modeling the relationship between protein activities of TFs regulating gene *n* and the variation of gene expression under sample *m* from its baseline expression $r{\text{'}}_{n}$, using a log-linear model^17,18^ as follows:

1 $$\frac{y{\text{'}}_{n,m}}{r{\text{'}}_{n}}=\prod _{t}{({x}_{t,m})}^{{a}_{{c}_{n},t}{b}_{{c}_{n},t}},$$

In time-course gene expression data, the baseline expression $r{\text{'}}_{n}$ refers to the expression value under time ‘0’; in steady-state data, $r{\text{'}}_{n}$ can be approximated using the mean expression across all samples and conditions. ${a}_{{c}_{n},t}$ represents the regulation effect of the binding event ${b}_{{c}_{n},t}$ of TF *t* in module *c*_*n*_. The regulation is effective when TF *t* physically binds to promoter or enhancer regions associated with the gene *n*. *x*_*t,m*_ represents the proteomic response (or activity) of TF *t* in sample *m*. The following equation can be derived from Eq. (1) after taking the logarithm:

2 $$\log (y{\text{'}}_{n,m})=\sum _{t}{a}_{{c}_{n},t}{b}_{{c}_{n},t}\,\log (x{\text{'}}_{t,m})+\,\log (r{\text{'}}_{n}).$$

We define ${y}_{n,m}=\,\log (y{\text{'}}_{n,m})$, ${x}_{t,m}=\,\log (x{\text{'}}_{t,m})$, ${r}_{n}=\,\log (r{\text{'}}_{n})$ and add ${e}_{n,m}$ to denote the noise in the gene expression data. Then, we derive the following log-linear model:

3 $${y}_{n,m}=\sum _{t}{a}_{{c}_{n},t}{b}_{{c}_{n},t}{x}_{t,m}+{r}_{n}+{e}_{n,m}.$$

The log linear model has been used previously to describe the relationship between promoter activity and transcription factor activities^17^. In particular, the value of ${a}_{{c}_{n},t}$ is determined by the Hill coefficients and the TF binding affinity to the promoter region^18^. Model parameters of ${x}_{t,m}$, ${a}_{{c}_{n},t}$ and ${b}_{{c}_{n},t}$ are dependent with each other. By estimating all model parameters we can eventually identify module *c*_*n*_.

When multiple TFs are jointly studied, unlike many existing tools that only report strong regulators for each gene^19–21^, which leads to an incomplete reconstruction of the true regulatory network, BICORN lowers the model overfitting effects as follows: it introduces an index variable *c*_*n*_ linking a candidate CRM (a row in matrix **b**) to gene *n*. Regulation strengths ${a}_{{c}_{n},t}$ of all TFs in *c*_*n*_ are jointly estimated, including both strong and weak regulators (if any). Since the number of TFs in each *c*_*n*_ is relatively small (usually less than six), in every evaluation the overfitting effects are largely alleviated. After evaluating all candidate modules for gene *n* and repeating this process for every gene, in the end, a regulatory network with both strong and weak bindings is reconstructed. BICORN uses a hierarchical Bayesian framework to model dependencies between variables, which are robustly estimated using Gibbs sampling.

#### A hierarchical Bayesian framework

**Figure 2:**
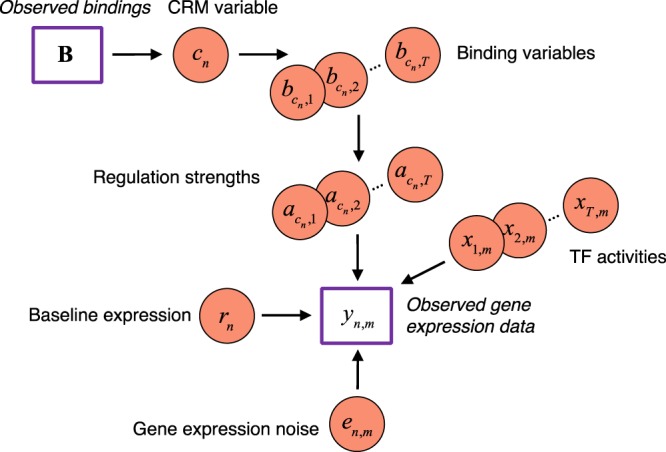
A hierarchical Bayesian framework for gene regulation modelling. CRM variables define the binding states of each gene. For each binding event, the regulation strength is either positive or negative to denoting gene activation or depression by the binding TF, respectively. Meanwhile, through TF-gene regulation, the TF activity is directly connected to target gene expression, along with residual baseline expression and noise.

Model variables included regulation strength matrix ${\bf{A}}=[{a}_{{c}_{n},t}]$, TF activity matrix ${\bf{X}}=[{x}_{t,m}]$, module index vector ${\bf{C}}=[{c}_{n}]$, residual baseline expression vector ${\bf{R}}=[{r}_{n}]$ and gene expression noise matrix ${\bf{E}}=[{e}_{n,m}]$. Given the log value of gene expression in matrix ${\bf{Y}}=[{y}_{n,m}]$ and prior TF-gene binding events in matrix ${\bf{B}}=[{b}_{{c}_{n},t}]$, we proposed the herachical Bayesian framework shown in Fig. 2 to model dependencies between variables.

Based on this model, we define a posterior probability as:

4 $$\begin{array}{c}P({\bf{A}},{\bf{C}},{\bf{X}},{\bf{R}},{\sigma }^{2}|{\bf{Y}},{\bf{B}})\\ \propto P({\bf{Y}}|{\bf{B}},{\bf{A}},{\bf{C}},{\bf{X}},{\bf{R}},{\sigma }^{2})P({\bf{A}}|{\bf{C}})P({\bf{C}})P({\bf{X}})P({\bf{R}})P({\sigma }^{2})\\ \propto \prod _{n}\prod _{m}\prod _{t}P({y}_{n,m}|{a}_{n,t},{c}_{n},{x}_{t,m},{r}_{n},{\sigma }_{\varepsilon }^{2})P({a}_{n,t}|{b}_{{c}_{n},t})P({c}_{n})P({x}_{t,m})P({r}_{n})P({\sigma }_{\varepsilon }^{2})\end{array}.$$

To estimate these variables using Bayesian inference, we made proper prior distribution assumptions on each. The binary binding state *b* is defined by the CRM index variable *c*, which corresponds to a row of the CRM matrix ***B***. We assume a uniform prior on *c* ($P(c)=1/K$). The regulation strength variable *a* can be either positive (a TF activates gene expression) or negative (a TF suppresses gene expression) and is conditioned on *b*: If $b=1$, we assume a zero-mean prior Gaussian distribution on *a* as $P(a|b=1)=N(0,{\sigma }_{a,hyper}^{2})$ by assuming that in the whole regulatory network, regulation strengths *a* can be either positive or negative without informative prior; if $b=0$, there is no functioning regulatory relationship between the pair TF and gene so we set $a=0$.

As the mRNA expression of a TF can be either up-regulated or down-regulated with respect to the baseline expression, its proteomic response can also be positive or negative compared to the baseline activity. Therefore, we model TF activity as a Gaussian random process and each variable *x* follows a Gaussian distribution with prior $P(x)=N(0,{\sigma }_{x,hyper}^{2})$.

Variable *r* represents the baseline expression and is invariant across samples. The baseline expression arocess genes vary a lot. To infer model parameters jointly for all genes, gene expression data is log-transformed and then normalized with mean expression of each gene to zero. Theoritically, the baseline expression of each gene is zero. However, in real data process, the normalzaition process may not be perfect so we use variable *r* to denote the baseline expression residual. The gene expression noise *e* varies across samples. We assumed zero-mean Gaussian priors on both *r* and *e* as $P(r)=N(0,{\sigma }_{r,hyper}^{2})$ and $P(e)=N(0,{\sigma }_{e}^{2})$.

In this model, ${\sigma }_{x,hyper}^{2}$, ${\sigma }_{a,hyper}^{2}$ and ${\sigma }_{r,hyper}^{2}$ are hyperparameters whereas the noise level ${\sigma }_{e}^{2}$ is a variable because the gene expression data quality varies case by case. To ensure the convergence of Gibbs Sampling, we assume the conjugate prior of Gaussian——the inverse Gamma distribution on ${\sigma }_{e}^{2}$: $P({\sigma }_{e}^{2})=inverseGamma(\alpha ,\beta )$, with hyperparameters $\alpha$ and $\beta$. Hyperparameter selection is discussed in ***Supplementary Methods***.

#### Gibbs Sampling

Using Gibbs sampling, BICORN iteratively learns the marginal distribution of each variable to approximate the joint posterior distribution. The uncertainty of the observed gene expression measure ${y}_{n,m}$ is due to noise, which, conditioned on the other variables, is estimated as: ${e}_{n,m}={y}_{n,m}-\sum _{t}{a}_{{c}_{n},t}{b}_{{c}_{n},t}{x}_{t,m}-{r}_{n}$. As ${e}_{n,m}$ is a Gaussian distributed variable, the conditional probability $P({y}_{n,m}|{a}_{n,t},{c}_{n},{x}_{t,m},{r}_{n},{\sigma }_{\varepsilon }^{2})$ in Eq. (2) is a Gaussian distribution. Given that we assume Gaussian priors on TF activity *x*, regulation strength *a*, and baseline expression residual *r*, the marginal distribution of each variable is also Gaussian. Therefore, we sample these variables according to their posterior Gaussian distributions as follows:

5 $${x}_{t,m}\sim N({\mu \text{'}}_{x},{\sigma \text{'}}_{x}^{2}),$$

6 $${a}_{n,t}\sim N({\mu \text{'}}_{a},{\sigma \text{'}}_{a}^{2}),$$

7 $${r}_{n}\sim N({\mu \text{'}}_{r},{\sigma \text{'}}_{r}^{2}),$$

where the posterior Gaussian distribution parameters ${\mu \text{'}}_{x}$, ${\sigma \text{'}}_{x}^{2}$, ${\mu \text{'}}_{a}$, ${\sigma \text{'}}_{a}^{2}$, ${\mu \text{'}}_{r}$ and ${\sigma \text{'}}_{r}^{2}$ are updated after each round of sampling, as described in ***Supplementary Methods***. The CRM variable *c*_*n*_ is sampled according to the posterior probability:

8 $$p({c}_{n}=k)=\frac{P({c}_{n}=k|{\bf{Y}},{\bf{B}},{\bf{A}},{\bf{X}},{\bf{R}},{\sigma }_{e}^{2})}{\sum _{j}P({c}_{n}=j|{\bf{Y}},{\bf{B}},{\bf{A}},{\bf{X}},{\bf{R}},{\sigma }_{e}^{2})}.$$

The overall performance of sampled variables is controlled by the noise variance ${\sigma }_{\varepsilon }^{2}$, which, after each round of sampling over the other variables, is updated by sampling according to the conditional probability:

9 $${\sigma }_{e}^{2} \sim {\rm{inverse}}Gamma(\alpha \text{'},\beta \text{'}),$$

where $\alpha \text{'}$ and $\beta \text{'}$ are the posterior inverse Gamma distribution parameters and updated after each round of sampling. The detailed calculation of each parameter is described in ***Supplementary Methods***.

Using Gibbs sampling, BICORN iteratively estimated regulation strength, TF activity and CRMs (Fig. 1B). To initiate the sampling process, for each gene, based on its prior binding events and candidate module matrix, we randomly select a module whose binding events are all observed at the regulatory region of the current genes. We then randomly generate initial values of regulation strengths according to the prior Gaussian distribution. Similarly, for TF activities, we generate their initial Gaussian-distributed activities. The initial baseline expression residual can then be obtained using ${r}_{n}=\sum _{m}({y}_{n,m}-\sum _{t}{a}_{{c}_{n},t}{b}_{{c}_{n},t}{x}_{t,m})/M$ to ensure that the gene expression noise has a zero mean. We run Gibbs sampling to generate samples of each variable iteratively. As a carefully designed MCMC process, the sampling process should converge from different initial states to the final steady state. Therefore, we monitor the convergence of each variable based on the ratio of the within-sequence-variance and the between-sequence-variance for multiple sampling sequences, each starting from a different initial state^22^. When the sampler begins to converge to the stationary distribution, this ratio would be around 1. We monitor the sampling convergence for TF activities and regulation strengths. Once both of these converge, we accumulate samples on CRMs for each gene.

### BICORN output

The gene sampling frequencies for individual CRMs represent the posterior probabilities of regulation. Using a threshold probability (i.e. 0.85) as a cutoff, genes for each CRM are identified and further CRMs are ranked by the number of target genes each regulates (Fig. 1C). Meanwhile, for each TF, its sampling frequency (summed over all CRMs containing this TF) represents the posterior probability of TF-gene regulation (Fig. 1D).

## Results

### BICORN accurately infers CRMs, genes and regulatory networks

We tested and compared the performance of BICORN and those of integrative Bayesian tools BNCA^23^ and COGRIM^12^ on simulated data. We simulated ten different TF-gene binding networks including 160 genes and 20 TFs, each with 80 true target genes. Each gene was regulated by a CRM with 2 ~ 6 TFs. Regulation strengths were generated as zero-mean and one-standard deviation Gaussian random variables with a high score (>2 or < −2) for strong regulators and a low but non-zero score for weak regulators. TF activities were simulated for 20 TFs using Gaussian random processes with zero-mean and one-standard deviation. Given the simulated network and variable values, a gene expression dataset was simulated using the log-linear model as in Eq. (1). We added 30% false positive interactions to each simulated network and used the perturbed bindings as prior TF-gene binding events. We also added zero-mean Gaussian distributed noise to the simulated gene expression data with a variance of 0.5 (signal-to-noise ratio 3 dB).

**Figure 3:**
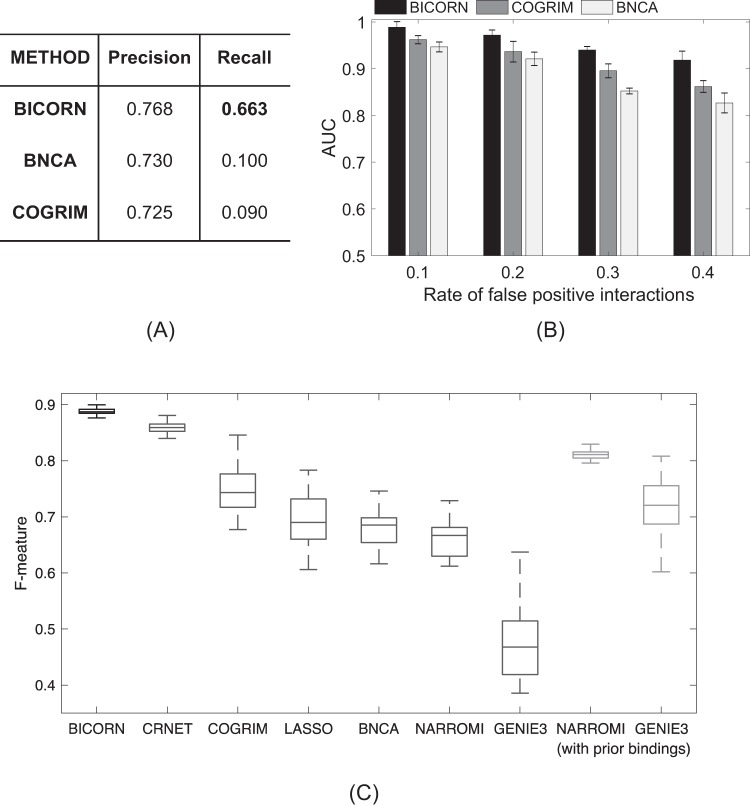
Performances of BICORN and competing methods on simulated and benchmark datasets. (**A**) Precision-recall performances on CRM identification using simulated data. (**B**) AUC performances on target gene prediction using simulated data. (**C**) F-measure performances on TF-gene regulatory network identification using DREAM 4 in silico benchmark data. Central values of the box plot represent the median, the box extends from quantile 25 to the 75 percentiles, and whiskers extend to the maximum and minimum values no further than 1.5 times the interquartile range from the hinge.

We first calculated precision and recall of each method on CRM identification. As shown in Fig. 3A, achieving the same precision performance around 0.75, BICORN correctly identified CRMs for more genes, with a recall of 0.66, compared to the recall values of ~0.1 for both BNCA and COGRIMN. BICORN also identified more target genes. In many situations, given data of TF-gene regulation and gene expression, genes are major targets for identification. Here, a target gene was correctly identified when at least one of its upstream regulators was captured. We simulated more scenarios by adding different levels (10% ~ 40%) of false positive interactions to the prior binding networks. As shown in Fig. 3B, BICORN exhibited superior performance.

To further evaluate the performance of BICORN on regulatory network inference (recovering TF-gene binding events), we downloaded benchmark networks and training time course gene expression data from the Synapse platform (https://www.synapse.org/, Synapse ID: syn3049712), which were used for DREAM challenge 4^24^. There were 5 different benchmark regulatory networks and 50 time-course gene expression datasets (10 for each network). The network was very sparse with a median of two TFs regulating each gene. To evaluate the robustness of BICORN on regulatory network inference, we perturbed prior binding observations by introducing 5% to 50% false negative interactions (a ‘true’ TF-gene interaction in the benchmark network but ‘missed’ by the prior binding matrix) or 5% to 100% false positive interactions. The F-measure (2*precision*recall/(precision+recall)) of BICORN is shown in Supplementary Fig. S1. When the rate of false negative interactions is under 15% or the rate of false positive interactions is under 30%, BICORN can achieve an average F-measure higher than 0.9.

We further compared the performance of BICORN to that of existing methods. Besides the above mentioned Bayesian methods, we included additional competing methods including CRNET^20^, LASSO^19^, NARROMI^25^ and GENIE3^26^. CRNET and LASSO are integrative methods using prior TF-gene bindings and gene expression. NARROMI and GENIE3 are inferring gene regulatory networks using gene expression data only. Specific for NARROMI and GENIE3, for a fair compare we checked their accuracy respectively using all the originally inferred bindings or inferred bindings with prior observations only, in which the prior knowledge was introduced to the model. We randomly perturbed benchmark networks by adding 15% false negative connections and 30% false positive connections and used the perturbed network as input. F-measure of each method under default setting was recorded. BICORN performed better than all selected competing methods and overperformed NARROMI and GENIE3 with prior TF-gene bindings provided (Fig. 3C).

### CRM at promoters and enhancers of breast cancer cells

The chromatin states of enhancers and promoters exhibit different histone markers^27^. Using BICORN, we further investigated these differences by studying CRMs at enhancers and promoters and comparing functional TFs at these regions. We applied BICORN to data acquired from breast cancer MCF-7 cells: 39 TFs ChIP-seq profiles from ENCODE (https://www.encodeproject.org/) and two 17b-E2 treated MCF-7 RNA-seq datasets from the GEO database (https://www.ncbi.nlm.nih.gov/geo/, with accession numbers GSE62789 and GSE51403). The GSE62789 dataset included ten samples measured within 24 hours (hrs) of 10 nM 17b-E2 treatment. The GSE51403 dataset included seven samples measured under vehicle condition and another seven samples measured after 24 hrs treatment of 10 nM 17b-E2. We collected differentially expressed genes as reported in the original publications^28,29^ and identified 235 common E2 active genes for further exploration. To test the robustness of CRMs predicted by BICORN, we respectively applied BICORN to the above two relevant gene expression datasets and characterized genes showing significant differential expression in both datasets.

#### Promoter analysis

**Figure 4:**
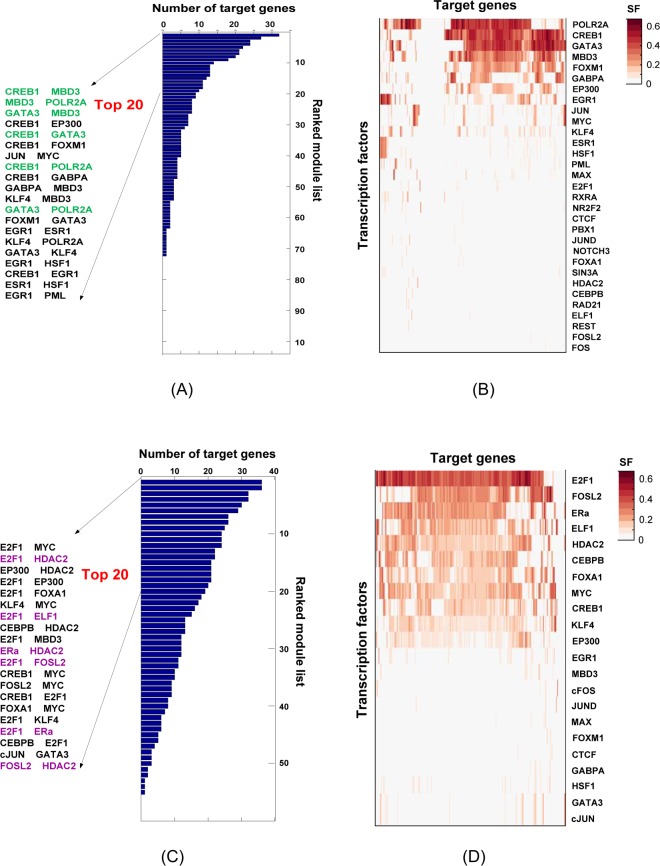
TFs functional at promoter or enhancer regions of E2 responsive target genes in breast cancer MCF-7 cells. (**A**) BICORN identified top 20 CRMs functioning at promoter regions; (**B**) strong (green), weak (light green) and little-active (grey) TFs at promoter regions; (**C**) BICORN identified top 20 CRMs functioning at enhancer regions; (**D**) strong (purple), weak (light purple) and little-active (grey) TFs at enhancer regions.

We annotated peaks of each TF using GREAT^30^ and selected peaks falling in promoter regions (+/1k bps around transcription start sites). Based on the binding events (annotated peaks) of 39 TFs on 235 active genes, BICORN identified 73 candidate CRMs. Further integrating the prior TF-gene binding events with each of the above mentioned target gene expression dataset, BICORN finally inferred 549 reliable CRM-gene interactions (sampling frequency> 0.85) for the GSE62789 dataset and 545 interactions for the GSE51403 dataset, with an overlap of 466 (86%) CRM-gene interactions covering 32 TFs and 113 target genes. Learned distributions of model parameters are shown in Supplementary Fig. S2, in which the posterior regulatory strengths, TF activities and residuals of baseline expression all follow Gaussian distributions. Focusing on those common interactions, CRMs were sorted based on the number of target genes regulated by each and the top 20 CRMs were listed in Fig. 4A. Moreover, all TFs were sorted using TF-gene interactions, as shown in Fig. 4B. We found that MBD3 frequently interacted with other TFs, for example POLR2A, at promoter regions. As a member of the nucleosome remodeling and deacetylation (NuRD) complex, MBD3 was demonstrated to be enriched at active promoters^31^ and to influence the association of POLR2A at transcription start sites^32^.

#### Enhancer analysis

To associate TF bindings at enhancer regions with target genes, we downloaded MCF-7-specific enhancer-like regions and ChIA-PET data from the ENCODE database. TF peaks were first aligned to enhancer regions and then looping to genes using ChIP-PET chromatin interactions. Distal binding events of the 39 TFs, 1857 enhancers and 235 target genes were collected, based on which BICORN identified 56 candidate CRMs. Integrating the distal TF binding events associated with genes and target gene expression together, BICORN finally identified 822 distal CRM-gene interactions using the GSE62789 dataset and 816 interactions using the GSE51403 dataset, with 630 (77%) common CRM-gene interactions covering 22 TFs and 99 target genes. Learned distributions of model parameters are shown in Supplementary Fig. S2, in which the posterior regulatory strengths, TF activities and residuals of baseline expression all follow Gaussian distributions. The top 20 CRMs and sorted TFs were shown in Fig. 4C,D, respectively. Among these, E2F1 actively binds at enhancer regions and interacts with factors including EP300, a well-known enhancer factor^33^. The loss of E2F1 did not change expression of known E2F target genes, suggesting that perhaps E2F1-specific regulatory regions are distinct from known E2F target promoters^34^. Indeed, in our analysis, E2F1 is active enriched in enhancer regions and associates with several other TFs by forming multiple CRMs, but much less active at promoter regions.

Comparing functional TF bindings at promoter and enhancer regions we found that, in general, active TFs at promoter and enhancer regions differ in this breast cancer study (Fig. S1). HSF1, cJUN, EGR1, GABPA, FOXM1, MBD3 and GATA3 had intensive bindings at gene promoters but almost none at enhancers. Another six TFs including E2F1, FOSL2, ELF1, HDAC2, CEBPB and FOXA1 were highly active at enhancers with few functional bindings at promoters. Only four TFs functioned at both regions but either individual enrichment or associations among each other were quite different between enhancers and promoters. Therefore, CRMs should be studied separately at promoters and enhancers to reveal a complete map of regulatory signals mediating gene transcription.

### Functional CRMs identification of diverse cell types

To demonstrate BICORN’s broad applicability, we applied it to six other cell types: K562, GM12878, HepG2, A549, SK-N-SH and HCT116. For each cell type, TF ChIP-seq profiles were downloaded from the ENCODE database and a matched gene expression data set was downloaded from the GEO database, as summarized in Table 1. Detailed data processing process as well as the number of enhancers, genes and modules studied for each case can be found from ***Supplementary Results***.

**Table 1:** Prior bindings and gene expression data used for CRM inference.

Cell line	Number of TFs (ENCODE database)	Gene expression data (GEO database)	Promoter	Enhancer
*K562*	203	*GSE1036*	✓	✓
*GM12878*	122	*GSE51709*	✓	✓
*HepG2*	108	*GSE6869*	✓	✓
*A549*	52	*GSE69667*	✓	
*SK-N-SH*	28	*GSE9169*	✓	
*HCT116*	20	*GSE14103*	✓	✓

**Figure 5:**
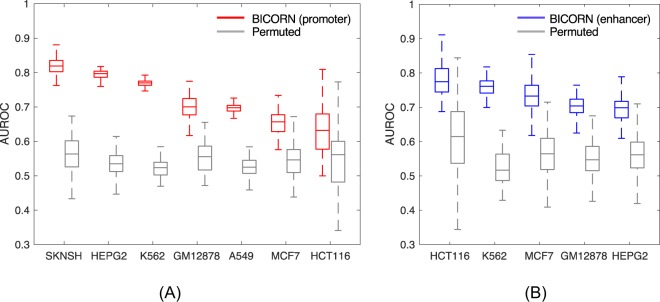
Prediction performance on cell type-specific promoter or enhancer activities using BICORN inferred CRMs. (**A**) promoter prediction for seven cell types; (**B**) enhancer prediction for five cell types. Central values of the box plot represent the median, the box extends from quantile 25 to the 75 percentiles, and whiskers extend to the maximum and minimum values no further than 1.5 times the interquartile range from the hinge.

TFs functionally activate/suppress the regulatory activity of the region that they bind to. For each cell type, we validated the cell type-specificity of BICORN inferred TF modules by checking their regression performance to the matched promoter or enhancer activities. Cell type-specific promoter or enhancer activities for regions studied by BICORN were downloaded from the FANTOM5 database^35^. For each cell type, we built a logistic regression model using 70% active/inactive promoter or enhancer regions with BICORN inferred CRMs or with randomly permuted CRMs. Active/inactive promoter or enhancer region selection is described in ***Supplementary Results***. AUROC (area under receiver operating characteristic curve) is calculated to evaluate the prediction performance of the built model on the 30% hold-out regions (Fig. 5A for promoter and Fig. 5B for enhancer). In all studied cases, the performance of BICORN inferred CRMs is significantly higher than that of the permuted CRMs (Wilcoxon rank sum test *p*-value <1e-9). Numbers of active/inactive promoter and enhancers in each cell type and the significance p-value of each case can be found in Supplementary Table S1.

Further examination of CRMs predicted by BICORN revealed strong and consistent cell-type specificity to existing literatures. At promoters, CTCF-RAD21 was previously reported to be functional around transcription start sites of genes specific to K562 cells with evidence suggesting that RAD21 contributes to stable CTCF bindings^36^. SOX13-SOX5 was identified from HepG2 promoter study. Both transcription factors are from SOX family and SOX13 complements SOX5 functionally^37^. In A549 cells, the association between E2F6 and MAX was top ranked by BICORN. MAX is a member of the E2F6 complex, which usually binds to E2F-resposive promoters, and is involved in gene repression^38^. In SK-N-SH cells, one of the top-ranked CRMs included three transcription factors: CTCF, RAD21, and SMC3. CTCF and the cohesin complex, consisting of the core subunits SMC3 and RAD21, were found to colocalize extensively throughout mammalian genomes^39^. In HCT116 cells, the CRM of FOSL1-JUND was identified. To regulate gene transcription, FOSL1 requires a dimerization partner, which is often a member of the JUN family^40^.

At enhancers, NRF1-RFX1 was the top-ranked CRM in K562 cells with RFX1 exhibiting RNA polymerase II distal enhancer sequence-specific binding^41^. In another blood cell type, GM12878, ELF1-ZEB1 was highly ranked with two B-cell specific transcription factors: MTA2-TBX21, which was predicted to be active at its enhancers, and with MTA2 occupancy on enhancers demonstrated in^42^. At HepG2 enhancers, BICORN found a strong association between SUZ12 and ZNF143–two PRC2 epigenomic signatures, whose association with enhancers was previously reported^43^. In HCT116 cells, BICORN identified POLR2A-YY1, which generally occupies active enhancers and promoters across cell types and plays structural roles in enhancer-promoter loops^44^.

## Discussion

Identifying high-order TF associations (as CRMs) and how these affect the gene transcription process is an open challenge. Conventional regulatory network analysis cannot tell how TFs associate with each other to regulate specific genes. This challenge is also hard to address experimentally as the simultaneous knockdown of more than one protein is not trivial. Our tool, BICORN, addresses a critical need: it enables to efficiently identify context-specific CRMs among hundreds of TFs. BICORN does this by integrating (proximal or distal) TF-gene binding events and target gene expression data and builds a hierarchical Bayesian framework to estimate values of all modelled variables. We have applied BICORN to both simulated and real data and have demonstrated its robustness and efficiency on module inference. BICORN is a general tool and can be readily applied to data generated from any cell type, tissue or organism. BICORN is freely available as open-source software.

## Supplementary information

Supplemental Material.
