# Author Correction: BICORN: An R package for integrative inference of *de novo* cis-regulatory modules

**DOI:** 10.1038/s41598-020-74149-y

**Published:** 2020-10-07

**Authors:** Xi Chen, Jinghua Gu, Andrew F. Neuwald, Leena Hilakivi-Clarke, Robert Clarke, Jianhua Xuan

**Affiliations:** 1 Bradley Department of Electrical and Computer Engineering, Virginia Polytechnic Institute and State University, 900 North Glebe Road, Arlington, VA 22203 USA; 2 Baylor Research Institute, 3310 Live Oak St, Dallas, TX 75204 USA; 3 Institute for Genome Sciences and Department Biochemistry and Molecular Biology, University of Maryland School of Medicine, Baltimore, MD 21201 USA; 4 Department of Oncology, Lombardi Comprehensive Cancer Center, Georgetown University Medical Center, 3970 Reservoir Road, Washington, DC 20057 USA

Correction to: *Scientific Reports* 10.1038/s41598-020-63043-2, published online 14 May 2020

The original version of this Article contained errors.

In the Abstract,

“Genome-wide transcription factor (TF) binding signal analyses reveal co-localization of TF binding sites based on inferred cis-regulatory modules (CRMs).”

now reads:

“Genome-wide transcription factor (TF) binding signal analyses reveal co-localization of TF binding sites, based on which cis-regulatory modules (CRMs) can be inferred.”

In addition, in the Methods section, under the subheading ‘BICORN input’,

“Binary TF-gene binding input is used because it is the signal format most commonly used by different resources.”

now reads:

“Binary TF-gene binding input is used because it is the signal format most commonly provided by different resources.”

Finally, the Acknowledgements section in this Article was incomplete.

“This work was supported by National Institutes of Health (NIH) grants CA149653 (to JX), CA164384 (to LHC) and CA149147 (RC), and by NIH-NIGMS grant R01GM125878 to AFN.”

now reads:

“This work was supported by National Institutes of Health (NIH) grants CA149653 (to JX), CA164384 (to LHC) and CA149147 (RC), and by NIH-NIGMS grant R01GM125878 to AFN. Note that open access publishing is supported by "VT Open Access Subvention Fund".”

These errors have now been corrected in the HTML and PDF versions of the Article.

