# Supplementary material for: BICORN: An R package for integrative inference of de novo cis-regulatory modules
Source: Sci Rep. 2020 May 14;10:7960. doi: 10.1038/s41598-020-63043-2 (PMC7224214; doi:10.1038/s41598-020-63043-2)
Supplement: Supplementary file 1 — Supplemental Material. [file 41598_2020_63043_MOESM1_ESM.docx]

# **BICORN: An R package for integrative inference of *de novo* cis-regulatory modules**

Xi Chen^1^, Jinghua Gu^2^, Andrew F. Neuwald^3^, Leena Hilakivi-Clarke^4^, Robert Clarke^4^, Jianhua Xuan^1,*^

^1^Bradley Department of Electrical and Computer Engineering, Virginia Polytechnic Institute and State University, 900 North Glebe Road, Arlington, VA 22203, USA.

^2^Baylor Research Institute, 3310 Live Oak St, Dallas, TX 75204, USA.

^3^Institute for Genome Sciences and Department Biochemistry & Molecular Biology, University of Maryland School of Medicine, Baltimore, MD 21201, USA.

^4^Department of Oncology, Lombardi Comprehensive Cancer Center, Georgetown University Medical Center, 3970 Reservoir Road, Washington, DC 20057, USA.

^*^ Corresponding author

*BICORN Supplementary File*

# **Supplementary Methods**

The posterior probability defined in Eq. (2) can be further extended as follows:

$\begin{matrix} P\left(\mathbf{A},\mathbf{C},\mathbf{X},\mathbf{R},\sigma^{2}\left|\mathbf{Y},\mathbf{B}\right.\right)=\prod_{n} \prod_{m} \frac{1}{\sigma_{e}}\exp\left(-\frac{1}{2\sigma_{e}^{2}}\left(y_{n,m}-\sum_{t} a_{c_{n},t}b_{c_{n},t}x_{t,m}-r_{n}\right)^{2}\right) \\ \times\prod_{n} \prod_{t} \frac{1}{\sigma_{a,hyper}}\exp\left(-\frac{1}{2\sigma_{a,hyper}^{2}}(a_{c_{n},t}b_{c_{n},t})^{2}\right) \\ \times\prod_{t} \prod_{m} \frac{1}{\sigma_{x,hyper}}\exp\left(-\frac{1}{2\sigma_{x,hyper}^{2}}x_{t,m}^{2}\right) \\ \times\prod_{n} \frac{1}{\sigma_{r,hyper}}\exp\left(-\frac{1}{2\sigma_{r,hyper}^{2}}r_{n}^{2}\right) \\ \times\left(\sigma_{e}^{2}\right)^{-\alpha_{hyper}-1}\exp\left(-\frac{\beta_{hyper}}{\sigma_{e}^{2}}\right). \end{matrix}$. (S-1)

**Step 1: Gibbs sampling of regulation strength** $a$

For each gene, if $b_{c_{n},t}=1$, we sampled $a_{c_{n},t}$ as follows:

$\begin{matrix} P\left(a_{c_{n},t}\left|b_{c_{n},t}=1,\mathbf{Y},\mathbf{B},\mathbf{A},\mathbf{C},\mathbf{X},\mathbf{R},\sigma^{2}\right.\right) \\ =\prod_{m} \frac{1}{\sigma_{e}}\frac{1}{\sigma_{a,hyper}}\exp\left(-\frac{1}{2\sigma_{e}^{2}}\left(y_{n,m}-\sum_{j\neq t} a_{c_{n},j}b_{c_{n},j}x_{j,m}-a_{c_{n},t}x_{t,m}-r_{n}\right)^{2}-\frac{1}{2\sigma_{a,hyper}^{2}}a_{c_{n},t}^{2}\right) \\ \propto\prod_{m} \frac{1}{\sigma_{e}}\frac{1}{\sigma_{a,hyper}}\exp\left(-\frac{x_{t,m}^{2}\sigma_{a,hyper}^{2}+\sigma_{e}^{2}}{2\sigma_{e}^{2}\sigma_{a,hyper}^{2}}a_{c_{n},t}^{2}+a_{c_{n},t}\frac{x_{t,m}}{\sigma_{e}^{2}}\left(y_{n,m}-\sum_{j\neq t} a_{c_{n},j}b_{c_{n},j}x_{j,m}-r_{n}\right)\right) \\ \propto\exp\left(-a_{c_{n},t}^{2}\sum_{m} \frac{x_{t,m}^{2}\sigma_{a,hyper}^{2}+\sigma_{e}^{2}}{2\sigma_{e}^{2}\sigma_{a,hyper}^{2}}+a_{c_{n},t}\sum_{m} \frac{x_{t,m}}{\sigma_{e}^{2}}\left(y_{n,m}-\sum_{j\neq t} a_{c_{n},j}b_{c_{n},j}x_{j,m}-r_{n}\right)\right) \end{matrix}$. (S-2)

From Eq. (S-2) it can be found that the posterior distribution of $a_{c_{n},t}$ was still a Gaussian distribution with mean and variance parameters as follows:

$\left\{\begin{matrix} \mu'_{a}=\frac{\sigma_{a,hyper}^{2}\sum_{m} (y_{n,m}-\sum_{j\neq t} a_{c_{n},j}b_{c_{n},j}x_{j,m}-r_{n})x_{t,m}}{\sigma_{a,hyper}^{2}\sum_{m} x_{t,m}^{2}+M\sigma_{e}^{2}} \\ \sigma'_{a}^{2}=\frac{\sigma_{a,hyper}^{2}M\sigma_{e}^{2}}{\sigma_{a,hyper}^{2}\sum_{m} x_{t,m}^{2}+\sigma_{e}^{2}M} \end{matrix}\right.$. (S-3)

We need to iteratively sample each $a_{c_{n},t}$ for $t=1\sim T$ because the above probability was conditional on the other $a_{c_{n},j}(j\neq t)$. We assigned non-informative prior on $a_{c_{n},t}$ by setting $\sigma_{a,hyper}^{2}=100$ to assume no knowledge of the regulation strength and let the sampler learn the distribution of true values of $a_{c_{n},t}$**.**

**Step 2: Gibbs sampling of TF protein response (activity)** $x$

$\begin{matrix} P\left(x_{t,m}\left|\mathbf{Y},\mathbf{B},\mathbf{A},\mathbf{C},\mathbf{X},\mathbf{R},\sigma^{2}\right.\right) \\ \propto\prod_{n} \frac{1}{\sigma_{e}}\frac{1}{\sigma_{x,hyper}}\exp\left(-\frac{1}{2\sigma^{2}}\left(y_{n,m}-\sum_{j\neq t} a_{c_{n},j}b_{c_{n},j}x_{j,m}-a_{c_{n},t}b_{c_{n},t}x_{t,m}-r_{n}\right)^{2}-\frac{1}{2\sigma_{x,hyper}^{2}}x_{t,m}^{2}\right) \\ \propto\prod_{n} \frac{1}{\sigma_{e}}\frac{1}{\sigma_{x,hyper}}\exp\left(-\frac{a_{c_{n},t}^{2}b_{c_{n},t}^{2}\sigma_{x,hyper}^{2}+\sigma_{e}^{2}}{2\sigma_{e}^{2}\sigma_{x,hyper}^{2}}x_{t,m}^{2}+x_{t,m}\frac{a_{c_{n},t}b_{c_{n},t}}{\sigma_{e}^{2}}\left(y_{n,m}-\sum_{j\neq t} a_{c_{n},j}b_{c_{n},j}x_{j,m}-r_{n}\right)\right) \\ \propto\exp\left(-x_{t,m}^{2}\sum_{n} \frac{a_{c_{n},t}^{2}b_{c_{n},t}^{2}\sigma_{x,hyper}^{2}+\sigma_{e}^{2}}{2\sigma_{e}^{2}\sigma_{x,hyper}^{2}}+x_{t,m}\sum_{m} \frac{a_{c_{n},t}b_{c_{n},t}}{\sigma_{e}^{2}}\left(y_{n,m}-\sum_{j\neq t} a_{c_{n},j}b_{c_{n},j}x_{j,m}-r_{n}\right)\right) \end{matrix}$. (S-4)

Eq. (S-4) revealed that the posterior distribution of $x_{t,m}$ was also a Gaussian distribution with mean and variance parameters as follows:

$\left\{\begin{matrix} \mu'_{x}=\frac{\sigma_{x,hyper}^{2}\sum_{n} (y_{n,m}-\sum_{j\neq t} a_{c_{n},j}b_{c_{n},j}x_{j,m}-r_{n})a_{c_{n},t}b_{c_{n},t}}{\sigma_{x,hyper}^{2}\sum_{n} a_{c_{n},t}^{2}b_{c_{n},t}^{2}+\sigma_{e}^{2}N} \\ \sigma'_{x}^{2}=\frac{\sigma_{e}^{2}N\sigma_{x,hyper}^{2}}{\sigma_{x,hyper}^{2}\sum_{n} a_{c_{n},t}^{2}b_{c_{n},t}^{2}+\sigma_{e}^{2}N} \end{matrix}\right.$. (S-5)

Note: we iteratively sampled each $x_{t,m}$ because the above probability was also conditional on the other $x_{j,m}(j\neq t)$. We set an informative prior on $x$ with $\sigma_{x,hyper}^{2}=1$.

**Step 3: Gibbs sampling of baseline expression residue** $r$

$\begin{matrix} P\left(r_{n}\left|\mathbf{Y},\mathbf{B},\mathbf{A},\mathbf{C},\mathbf{X},\sigma^{2}\right.\right) \\ \propto\prod_{m} \frac{1}{\sigma_{e}}\frac{1}{\sigma_{r,hyper}}\exp\left(-\frac{1}{2\sigma_{e}^{2}}\left(y_{n,m}-\sum_{t} a_{c_{n},t}b_{c_{n},t}x_{t,m}-r_{n}\right)^{2}-\frac{1}{2\sigma_{r,hyper}^{2}}r_{n}^{2}\right) \\ \propto\prod_{m} \frac{1}{\sigma_{e}}\frac{1}{\sigma_{r,hyper}}\exp\left(-\frac{\sigma_{e}^{2}+\sigma_{r,hyper}^{2}}{2\sigma_{e}^{2}\sigma_{r,hyper}^{2}}r_{n}^{2}+\frac{1}{\sigma_{e}^{2}}r_{n}\left(y_{n,m}-\sum_{t} a_{c_{n},t}b_{c_{n},t}x_{t,m}\right)\right) \\ \propto\exp\left(-r_{n}^{2}\sum_{m} \frac{\sigma_{e}^{2}+\sigma_{r,hyper}^{2}}{2\sigma_{e}^{2}\sigma_{r,hyper}^{2}}+r_{n}\sum_{m} \frac{1}{\sigma_{e}^{2}}\left(y_{n,m}-\sum_{t} a_{c_{n},t}b_{c_{n},t}x_{t,m}\right)\right) \end{matrix}$. (S-6)

The posterior distribution of $r_{n}$ was a Gaussian distribution with mean and variance parameters as follows:

$\left\{\begin{matrix} \mu'_{\eta}=\frac{\sigma_{e}^{2}\sum_{m} (y_{n,m}-\sum_{t} a_{c_{n},t}b_{c_{n},t}x_{t,m})}{M(\sigma_{e}^{2}+\sigma_{r,hyper}^{2})} \\ \sigma'_{\eta}^{2}=\frac{\sigma_{e}^{2}\sigma_{r,hyper}^{2}}{\sigma_{e}^{2}+\sigma_{r,hyper}^{2}} \end{matrix}\right.$. (S-7)

We assumed non-informative prior on $r_{n}$ as it highly depended on the gene expression data normalization process. We set hyper-parameter $\sigma_{r,hyper}^{2}=100$.

**Step 4: Gibbs sampling of** $c_{n}$

$\begin{matrix} P\left(c_{n}\left|\mathbf{Y},\mathbf{B},\mathbf{A},\mathbf{X},\mathbf{R},\sigma_{e}^{2}\right.\right)\propto\prod_{t} \prod_{m} P(y_{n,m}|a_{n,t},c_{n},x_{t,m},r_{n},\sigma_{\varepsilon}^{2})P(a_{c_{n},t}|c_{n}) \\ =\prod_{t} \prod_{m} \frac{1}{\sigma_{e}}\exp\left(-\frac{1}{2\sigma_{e}^{2}}\left(y_{n,m}-\sum_{t} a_{c_{n},t}b_{c_{n},t}x_{t,m}-r_{n}\right)^{2}\right) \\ \times\prod_{t} \frac{1}{\sigma_{a,hyper}}\exp\left(-\frac{1}{2\sigma_{a,hyper}^{2}}(a_{c_{n},t}b_{c_{n},t})^{2}\right). \end{matrix}$. (S-8)

We calculated the above conditional probability for all $c_{n}\in\{1,2,...,K\}$ and then sample $c_{n}$ as Eq. (6).

**Step 5: Gibbs sampling of posterior noise variance** $\sigma_{e}^{2}$

$\begin{matrix} P\left(\sigma_{e}^{2}\left|\mathbf{Y},\mathbf{B},\mathbf{A},\mathbf{C},\mathbf{X},\mathbf{R}\right.\right) \\ \propto\prod_{n} \prod_{m} \left(\sigma_{e}^{-1}\right)\exp\left(-\frac{1}{2\sigma_{e}^{2}}\left(y_{n,m}-\sum_{t} a_{c_{n},t}b_{c_{n},t}x_{t,m}-r_{n}\right)^{2}\right)\times\left(\sigma_{e}^{2}\right)^{-\alpha-1}\exp\left(-\frac{\beta}{\sigma_{e}^{2}}\right) \\ =\prod_{n} \prod_{m} \left(\sigma_{e}^{2}\right)^{-\alpha-1/2-1}\exp\left(-\frac{1}{\sigma_{e}^{2}}\left(\beta+\left(y_{n,m}-\sum_{t} a_{c_{n},t}b_{c_{n},t}x_{t,m}-r_{n}\right)^{2}/2\right)\right) \end{matrix}$(S-9)

As inferred from Eq. (S-9) that the posterior distribution of $\sigma_{e}^{2}$ followed an inverse Gamma distribution with parameters as follows:

$\left\{\begin{matrix} \alpha'=\alpha+\frac{1}{2} \\ \beta'=\beta+\frac{1}{2MN}\prod_{n} \prod_{m} \left(y_{n,m}-\sum_{t} a_{c_{n},t}b_{c_{n},t}x_{t,m}-r_{n}\right)^{2} \end{matrix}\right.$. (S-10)

We set hyper-parameters $\alpha=1$and $\beta=1$to make the prior distribution of $\sigma_{e}^{2}$ non-informative.

# **Supplementary Results**

***K562***

A K562 microarray data set was downloaded from the GEO database (GSE1036). Expression data were analyzed by MAS 5.0. According to ^1^, 1,311 differentially expressed genes were selected. We downloaded K562-specific ChIP-seq peaks of 203 TFs. For the promoter study, we overlapped above peaks with the promoter region of differential genes and identified 68 candidate CRMs for further exploration. After 1000 rounds of sampling, candidate CRMs were prioritized by the number of regulated target genes (posterior probability threshold for confident CRM-gene regulations is 0.9), a set of top-ranked CRMs (21 CRMs, each regulating at least 10% of the original set of target genes) were identified.

For the enhancer study, we downloaded K562-specific enhancer-like regions and ChIA-PET data from the ENCODE database. We overlapped TF peaks with enhancer regions that can be mapped to differential genes through enhancer-promoter interactions (each interaction with at least two paired-end ChIA-PET reads). In total, we collected 2,135 enhancers regions, 607 genes and 82 candidate CRMs for further exploration.

***GM12878***

A GM12878 microarray data set was downloaded from the GEO database (GSE51709). Expression data were analyzed through Affymetrix Expression Console. According to ^2^, 696 differentially expressed genes were selected. We downloaded GM12878 specific ChIP-seq peaks of 122 TFs the ENCODE database. For promoter study, we overlapped above peaks with the promoter region of differential genes and identified 79 candidate CRMs for further exploration. After 1000 rounds of sampling, 17 confident CRMs were identified.

For the enhancer study, we downloaded GM12878-specific enhancer-like regions and ChIA-PET data from the ENCODE database. We overlapped TF peaks with enhancer regions which can be mapped to differential genes through enhancer-promoter interactions (each interaction with at least two paired-end ChIA-PET reads). In total, we collected 1571 enhancers regions, 381 genes and 68 candidate CRMs for further exploration. After 1000 rounds of sampling, 10 confident CRMs were identified.

***HepG2***

A HepG2 microarray data set was downloaded from the GEO database (GSE6869). Expression data were obtained by MAS 5.0. According to ^3^, 874 differentially expressed genes were selected. We downloaded HepG2 specific ChIP-seq peaks of 108 TFs the ENCODE database. For the promoter study, we overlapped above peaks with the promoter region of differential genes and identified 55 candidate CRMs for further exploration. After 1000 rounds of sampling, 12 confident CRM were identified.

For the enhancer study, we downloaded HepG2-specific enhancer-like regions and ChIA-PET data from the ENCODE database. We overlapped TF peaks with enhancer regions which can be mapped to differential genes through enhancer-promoter interactions (each interaction with at least two paired-end ChIA-PET reads). In total, we collected 1457 enhancers regions, 433 genes and 43 candidate CRMs for further exploration. After 1000 rounds of sampling, 8 confident CRMs were identified.

***A549***

An A549 RNA-seq data set was downloaded from the GEO database (GSE69667). Expression data was analyzed through RSEM. According to ^4^, 1,633 differentially expressed genes were selected. We downloaded A549 specific ChIP-seq peaks of 52 TFs from the ENCODE database. Due to lack of A549 specific enhancer-like regions in the ENCODE database, in this case we conducted CRM inference at gene promoter region only. We overlapped ChIP-seq peaks with the promoter region of selected 1,633 genes and finally identified 72 candidate CRMs for further exploration. After 1000 rounds of sampling, 13 confident CRMs were identified.

***SK-N-SH***

A SK-N-SH microarray data set was downloaded from the GEO database (GSE9169). Expression data was analyzed through Affymetrix Expression Console. According to ^5^, 362 differentially expressed genes were selected. We downloaded SK-N-SH specific ChIP-seq peaks of 52 TFs from the ENCODE database. Due to lack of SK-N-SH specific enhancer-like regions in the ENCODE database, in this case we conducted CRM inference at gene promoter region only. We overlapped ChIP-seq peaks with the promoter region of selected genes and finally identified 61 candidate CRMs for further exploration. After 1000 rounds of sampling, 14 confident CRMs were identified.

***HCT116***

A HCT116 microarray data set was downloaded from the GEO database (GSE14103). Expression data was analyzed through Affymetrix Expression Console. According to ^6^, 286 differentially expressed genes were selected. We downloaded HCT116 specific ChIP-seq peaks of 20 TFs from the ENCODE database. For the promoter study, we overlapped above peaks with the promoter region of differential genes and identified 55 candidate CRMs for further exploration. After 1000 rounds of sampling, 24 confident CRMs were identified.

For the enhancer study, we downloaded HCT116-specific enhancer-like regions and ChIA-PET data from the ENCODE database. We overlapped TF peaks with enhancer regions which can be mapped to differential genes through enhancer-promoter interactions (each interaction with at least two paired-end ChIA-PET reads). In total, we collected 366 enhancers regions, 110 genes and 44 candidate CRMs for further exploration. After 1000 rounds of sampling, 6 confident CRMs were identified.

***FANTOM5 cell type-specific enhancer and promoter selection***

We downloaded cell type-specific enhancer or promoter activities from the FANTOM5 database (<http://fantom.gsc.riken.jp/5/>) for seven cell types studied in this paper, including MCF7, K562, GM12878, HepG2, A549, SK-N-SH and HCT116. During the input data process of BICORN, each gene was associated with only one promoter region. Thus, we directly obtained the promoter activity given each gene name. But for enhancers, many genes are associated with more than one enhancer regions, which are physically approximal to each other and have highly correlated regulatory activities. Therefore, we assigned to each gene a single enhancer activity by taking the mean activity of all associated enhancers. We select active/inactive promoters using threshold TPM of 6, and active/inactive enhancers using threshold TPM of 1.

**Table S1** Summary of FANTOM5 cell type-specific promoter and enhancer prediction.

| **Cell line** | **Promoter** | | | **Enhancer** | | |
| --- | --- | --- | --- | --- | --- | --- |
|  | **Active** | **Inactive** | ***p*-value** | **Active** | **Inactive** | ***p*-value** |
| *MCF-7* | 55 | 66 | 5.41e-28 | 35 | 35 | 8.36e-31 |
| *K562* | 204 | 1081 | 2.56e-34 | 191 | 107 | 2.56e-34 |
| *GM12878* | 369 | 288 | 5.10e-34 | 60 | 160 | 3.08e-33 |
| *HepG2* | 142 | 397 | 2.56e-34 | 30 | 163 | 5.71e-31 |
| *A549* | 319 | 1170 | 2.56e-34 | -- | -- | -- |
| *SK-N-SH* | 43 | 172 | 2.56e-34 | -- | -- | -- |
| *HCT116* | 31 | 32 | 2.67e-10 | 9 | 47 | 5.19e-26 |

**Supplementary Figures**

(A)

(B)

**Figure S1.** Simulation performance of BICORN using prior binding networks with different levels of false positive or false negative interactions.

Promoter study using the time-course gene expression dataset GSE62789

(A) regulation strength (B) TF activity (C) Baseline expression

Promoter study using the steady state gene expression dataset GSE51403

(D) regulation strength (E) TF activity (F) Baseline expression

Enhancer study using the time-course gene expression dataset GSE62789

(G) regulation strength (H) TF activity (I) Baseline expression

Enhancer study using the steady state gene expression dataset GSE51403

(J) regulation strength (K) TF activity (L) Baseline expression

**Figure S2.** Verifying the distribution of sampled variables in breast cancer MCF-7 studies.

**Figure S3.** TFs functional at promoter or enhancer regions of E2 responsive target genes in breast cancer MCF-7 cells. (A) foreground (green) and background (grey) TFs at promoter regions; (B) foreground (purple) and background (grey) TFs at enhancer regions. Common foreground TFs at both types of regions are labeled as ‘red’.

**Supplementary References**

1 Addya, S. *et al.* Erythroid-induced commitment of K562 cells results in clusters of differentially expressed genes enriched for specific transcription regulatory elements. *Physiol Genomics* **19**, 117-130, doi:10.1152/physiolgenomics.00028.2004 (2004).

2 Su, D. *et al.* Interactions of chromatin context, binding site sequence content, and sequence evolution in stress-induced p53 occupancy and transactivation. *PLoS Genet* **11**, e1004885, doi:10.1371/journal.pgen.1004885 (2015).

3 De, S. *et al.* PCB congener specific oxidative stress response by microarray analysis using human liver cell line. *Environ Int* **36**, 907-917, doi:10.1016/j.envint.2010.05.011 (2010).

4 Chang, H. *et al.* Synergistic action of master transcription factors controls epithelial-to-mesenchymal transition. *Nucleic Acids Res* **44**, 2514-2527, doi:10.1093/nar/gkw126 (2016).

5 Nishida, Y. *et al.* Identification and classification of genes regulated by phosphatidylinositol 3-kinase- and TRKB-mediated signalling pathways during neuronal differentiation in two subtypes of the human neuroblastoma cell line SH-SY5Y. *BMC Res Notes* **1**, 95, doi:10.1186/1756-0500-1-95 (2008).

6 Mizuno, H., Nakanishi, Y., Ishii, N., Sarai, A. & Kitada, K. A signature-based method for indexing cell cycle phase distribution from microarray profiles. *BMC Genomics* **10**, 137, doi:10.1186/1471-2164-10-137 (2009).
